# SIMBA-GNN: mechanistic graph learning for microbiome prediction

**DOI:** 10.1038/s41540-025-00631-w

**Published:** 2025-12-12

**Authors:** Javad Aminian-Dehkordi, Mohammad Parsa, Andrew Dickson, Mohammad R. K. Mofrad

**Affiliations:** 1 Molecular Cell Biomechanics Laboratory, Departments of Bioengineering and Mechanical Engineering, University of California, Berkeley, CA USA; 2 Molecular Biophysics and Integrative Bioimaging Division, Lawrence Berkeley National Laboratory, Berkeley, CA USA

**Keywords:** Computational biology and bioinformatics, Microbiology, Systems biology

## Abstract

Predicting how gut microbial communities assemble and change requires models that capture the underlying mechanisms driving interspecies interactions, not just taxonomic correlations. We present SIMBA, a simulation-augmented graph neural network that integrates mechanistic insights from metabolic simulations with edge-aware graph transformers to predict microbial community composition. Using a high-fiber dietary cohort mapped to metabolic networks, we ran thousands of pairwise simulations to infer cross-feeding probabilities, pathway activity fingerprints, and microbe-microbe functional similarity. These signals instantiate a global microbe-metabolite-pathway graph for learning. A custom heterogeneous graph transformer incorporates scalar edge attributes into attention. It is trained through a multi-stage pipeline combining self-supervised learning, supervised pretraining on simulated graphs, and fine-tuning on experimental microbial abundance data. Each individual’s microbiome is represented as a sample-specific instantiation of the shared mechanistic graph derived from metabolic simulations, where only the set of microbes detected in that individual varies. SIMBA learns from this mechanistic prior to predict microbial presence and relative abundance across individuals, enabling hypothesis-driven exploration of microbial ecosystems.

## Introduction

The human gut microbiome is a dynamic ecosystem that influences host physiology and has been linked to various diseases, from metabolic disorders to inflammatory and neurodegenerative conditions^1–3^. These associations stem not from individual microbes in isolation, but from complex, context-dependent interactions within the microbial community. Deciphering these interactions is key to uncovering the mechanisms underlying microbiome-associated health outcomes and informing the design of targeted therapeutic strategies^4,5^.

The inherent complexity of microbiome data, often high-dimensional, compositional, and sparse, has driven the development of a diverse array of computational tools. Early approaches to microbiome analysis focused on identifying statistical associations, using co-occurrence networks to infer potential microbial relationships^6,7^. While useful for hypothesis generation, these correlation-based methods are limited in capturing the dynamic, nonlinear interactions, such as metabolic cross-feeding and competition, that shape microbial community behavior^8^. Subsequently, the field adopted traditional machine learning models, such as Random Forests and support vector machines, which have proven effective for predictive tasks like classifying disease states from taxonomic profiles^9,10^. However, these models typically treat microbial features as independent variables, overlooking the networked structure of the ecosystem, and their “black-box” nature can limit biological interpretability. More advanced deep learning architectures, including convolutional and recurrent neural networks, have shown promise in capturing nonlinear patterns but face challenges with small sample sizes common in microbiome studies, leading to risks of overfitting and a demand for large datasets that are often unavailable^11,12^.

To ground predictions in biological mechanisms, another class of methods uses metabolic networks, which are knowledge bases of all known biochemical reactions in an organism^13^. These “bottom-up" models stand in contrast to the “top-down" data-driven approaches mentioned above^14^. By integrating multi-omics data, genome-scale metabolic models (GEMs) can simulate metabolic fluxes to predict microbial growth and interactions under specific conditions^15^. Community-level GEMs model the metabolic interplay between species, offering a powerful way to study phenomena like cross-feeding^16^. However, translating the output of these complex simulations into accurate, community-wide predictions of microbial abundances remains a significant challenge, often limited by computational cost and uncertainty in model parameters.

Our work is situated at the confluence of these approaches, building upon recent advances in graph neural networks (GNNs). GNNs are designed to process data structured as graphs, making them theoretically ideal for analyzing the irregular and complex network data that define microbial ecosystems^17,18^. However, the utility of a GNN is fundamentally dependent on the underlying graph structure^19^. To date, most GNN applications in microbiome research have relied on the same correlation-based graphs used in early association studies^20,21^, which may not reflect true biological interactions and fail to incorporate known mechanistic constraints^22^. This limitation prevents them from leveraging the rich biological knowledge curated in models like GEMs.

**Fig. 1:**
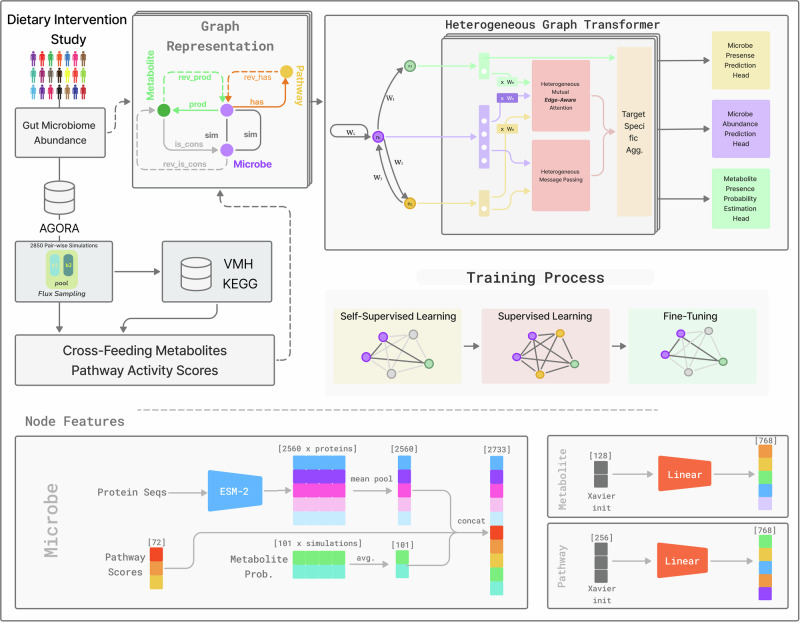
SIMBA for mechanistic microbiome prediction. Left—Data sources. A dietary intervention cohort of 186 individuals provides per-sample microbial relative abundances, mapped to their corresponding metabolic networks. 2850 pairwise simulations using flux sampling yield the core mechanistic data for our graph: the probabilities of metabolite cross-feeding and the metabolic pathway activity scores for each microbe. Middle-left—Graph construction. The simulation data were used to construct a single, global heterogeneous graph that serves as a mechanistic map of the ecosystem. The graph consists of three node types: microbe (purple), metabolite (green), and pathway (yellow), connected by seven directed edge types: (i) has/rev_has edges link microbes to their active pathways, (ii) bidirectional sim edges are added between pairs of microbes that exhibit high functional similarity (pathway score cosine similarity >0.85), representing their symmetric relationship, and (iii) prod, cons with their reverse edges, whose weights ${{we}={\log }({1+}|{\rm{flux}}|)}$ encode interaction strength. Middle-right—Heterogeneous graph transformer. Node features are first projected to 768-d and passed through three layers of our custom *edge-aware* HGT. Attention scores are modulated by the scalar edge weight *w*_*e*_. The model outputs feed three task-specific heads: a per-sample softmax abundance regressor (primary), a BCE presence classifier (auxiliary), and a BCE metabolite-probability estimator. Bottom—Training schedule. The network is optimized in three stages: (i) Self-supervised GraphCL to initialize embeddings, (ii) Supervised pretraining on simulated graphs with BCE, Tweedie (*p* = 1.5) and metabolite BCE losses, and (iii) Fine-tuning on experimental graphs using only BCE and Tweedie losses (ranking loss tested but not retained). Feature masking and edge dropout of 0.1 are applied throughout. Bottom-most—Node features. Microbe vectors concatenate (i) averaged 2560-d ESM-2 protein embeddings, (ii) 72-d log_1+_ pathway scores, and (iii) 101-d metabolite fingerprints, yielding 2733 features before projection. Metabolite and pathway nodes start from random 128-d and 256-d embeddings, respectively, that are linearly mapped to 768-d. Fine-tuning input. To predict the abundance profile for an individual sample, the model processes the entire global graph. Each experimental sample is represented as a per-sample instantiation of the shared global graph; a non-learned membership mask is applied only within the abundance head (softmax/loss) to target the calculation to the microbes present in that sample. This mask is not provided as an input feature to the GNN encoder, thus avoiding label leakage.

To address these challenges, we introduce SIMBA, a novel framework that bridges the gap between mechanistic simulation and deep learning by using pairwise GEM simulations to generate mechanistically grounded graphs (Fig. 1). These graphs explicitly encode metabolic dependencies, such as cross-feeding patterns, providing a biologically informed structure for the GNN. Each simulation yields a graph where nodes represent microbial species, metabolic pathways, and metabolites, and edges encode the direction, microbe similarity, and the probability of the metabolites being produced and consumed. Our custom-designed GNN learns from these graphs using a multi-stage pipeline of self-supervised learning, simulation-based pretraining, and fine-tuning on experimental microbial abundance data to predict community-level microbial abundances.

To ground this approach in real-world microbiome contexts, we use a dataset of fecal metagenomes from individuals on a high-fiber diet and simulate all pairwise interactions among microbes using their corresponding metabolic networks. These simulations generate a rich interaction dataset under diet-constrained conditions and enable the construction of labeled graphs that capture microbial cross-feeding dynamics. In practical terms, each sample analyzed by SIMBA-GNN is represented as a personalized version of a shared metabolic-interaction graph. The underlying topology encoding cross-feeding and pathway relationships remains fixed, while the subset of microbes observed in each individual defines the specific input instance. The model is trained to predict which microbes appear and in what proportions within this mechanistic context.

Our contributions are as follows:

- A novel computational pipeline that generates a large-scale dynamic dataset of pairwise microbial cross-feeding interactions using metabolic networks constrained by realistic dietary inputs.
- A custom GNN architecture tailored to learn from simulation-derived microbial interactions for the prediction of microbial abundances.
- Evaluation of the model’s predictive performance on microbial abundances against established GNN models, demonstrating the potential of integrating metabolic networks with advanced deep learning for understanding complex microbial ecosystems.

This study opens new avenues for personalized microbiome modeling and precision therapeutics by providing a scalable, mechanistic approach to understanding the human gut microbiome ecosystem.

## Results

### Construction of SIMBA framework

To build a predictive model grounded in biological mechanisms, we developed the SIMBA framework. This involved a multi-step process that began with simulating microbial metabolic interactions to create a biologically informed graph structure, which then served as the foundation for a custom graph neural network architecture (Fig. 1).

#### Mechanistic priors with metabolic simulations

The foundation for our graph is a rich dataset of potential metabolic interactions. Using 76 GEMs, we simulated 2850 pairwise co-culture under anaerobic conditions that mimicked a high-fiber diet. Flux sampling quantified metabolic exchanges and pathway-level activity profiles for each microbial pair. From the simulations, we extracted metabolite fingerprints-probabilities of metabolite cross-feeding-and pathway activity fingerprints summarizing flux through functional pathways. This simulation-based approach provides a mechanistic means of inferring ecological interactions that are often difficult to measure.

#### Graph neural network architecture

The simulation outputs were used to construct a global, heterogeneous graph representing the entire microbial ecosystem. The graph served as input to a custom edge-aware heterogeneous graph transformer (HGT) that embeds scalar edge attributes-reflecting flux magnitudes and metabolite-exchange probabilities-directly into the attention mechanism. Each node type (microbe, metabolite, pathway) was initialized with biologically meaningful features: concatenated protein-language embeddings, pathway fingerprints, and metabolite fingerprints for microbes, and trainable embeddings for pathways and metabolites. The network comprises three transformer layers (768-dimensional hidden states and 12 attention heads) with residual connections and dropout of 0.2.

#### Prediction objectives and workflow

Task-specific output heads predict microbial presence (binary), relative abundance (softmax-normalized), and metabolite presence probabilities (auxiliary task). Training proceeded through three stages, including self-supervised initialization, supervised pretraining on simulated graphs, and fine-tuning on experimental data, which allows transfer of mechanistic knowledge to observed communities. This construction forms the mechanistic foundation for the predictive analyses described below. The detailed simulation settings, loss-function definitions, and hyperparameter optimization procedures are provided in the Methods section.

### Characterization of microbial community data and baseline model performance

**Fig. 2:**
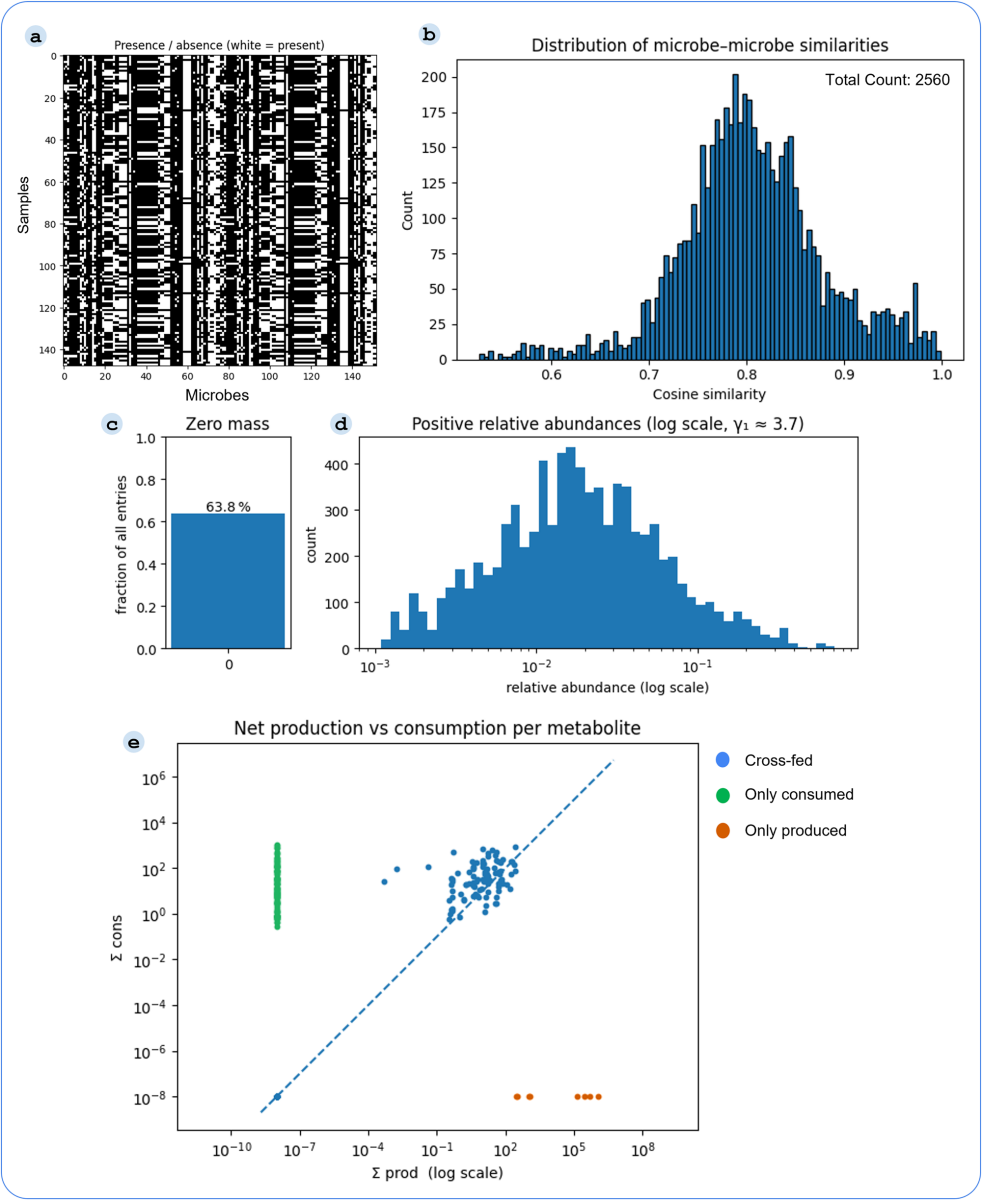
Microbial abundance patterns and metabolic-interaction landscape. **a** Presence/absence heatmap of microbes across samples, showing distinct distribution patterns. **b** Histogram of cosine similarities between pathway score profiles, indicating varying degrees of similarity across samples. **c** Fraction of zero entries (63.8%) in the microbial abundance matrix, illustrating data sparsity. **d** Distribution of nonzero relative abundances on a log scale, showing a heavy-tailed distribution with a shape parameter of 3.7. **e** Comparison of total simulated production versus consumption for each metabolite, which highlights potential metabolic sources and sinks in the microbial community.

Our approach to building a graphical representation of the gut microbiome for predictive modeling began with characterizing experimental samples and the outputs from pairwise metabolic simulations. These data were instrumental in shaping the structure and features of our heterogeneous graph. An overview of this foundational data characterization is presented in Fig. 2.

The microbial landscape within our samples exhibits clear presence and absence patterns for diverse microbes (Fig. 2a). Panels 2c, d further characterize these data, showing that 63.8% of abundance entries are zero and that the distribution of nonzero values follows a heavy-tailed log distribution. Understanding these abundance distributions is important, as they represent the primary predictive target for our models. Moreover, insights into metabolic interplay emerged from pairwise simulations. Figure 2e visually compares the net production and consumption capabilities per metabolite across all microbes in the community. This analysis reveals potential metabolic sources and sinks within the simulated ecosystems and forms the basis for the microbe-metabolite interaction edges in our graph.

Collectively, these analyses underscore the heterogeneity of microbial membership, the complexity of functional similarities, the challenging nature of abundance distributions, and the intricate web of simulated metabolic interactions. This rich, multimodal information landscape motivated the use of GNNs capable of integrating diverse data types to model microbial community behavior.

To establish a performance benchmark, we initially evaluated several established GNN architectures: SWM-GNN, GraphSAGE, MPGNN, and GNN. As illustrated in Supplementary Fig. 3, the models showed modest performance in predicting microbial abundances, with Spearman rank correlations generally below 0.6. This highlighted the limitations of standard GNNs for this complex task and motivated the development of our specialized SIMBA architecture.

### Performance across the training pipeline

To address the identified challenges, we propose SIMBA to predict microbial community composition. This model uses simulated metabolic cross-feeding interactions within an HGT framework to predict both microbial presence and relative abundances. The model’s performance was established through a three-stage training regimen-self-supervised learning, supervised pretraining, and fine-tuning, and its architecture was refined via systematic hyperparameter optimization.

#### Pretraining on simulated data

Following SSL, SIMBA was pretrained on a comprehensive dataset of simulated pairwise microbial interactions, enabling the model to learn the fundamental patterns of microbial metabolic cross-feeding. The convergence of the training loss over epochs is shown in Supplementary Fig. 4. The total training loss, alongside its components, BCE loss, Tweedie loss, and rank loss, all showed a consistent decrease over epochs of pretraining, indicating stable learning dynamics. For the auxiliary task of predicting metabolite cross-feeding presence, the model attained an accuracy of 0.96. The corresponding F1-score and recall for this task were 0.83 and 0.72, respectively, which further supports the model’s capability to identify these interactions in the simulated data.

Collectively, the results from the supervised pretraining stage indicate that the model successfully learned to model key aspects of microbial ecology within the context of the simulated data. This provided a strong foundation for the subsequent fine-tuning stage on experimental data.

#### Fine-tuning on experimental data

The final stage involved fine-tuning the pretrained SIMBA model using experimental microbial abundance data. This allowed the model to adapt its learned representations and predictive capabilities to real-world community contexts. The fine-tuning loss progression is presented in Supplementary Fig. 5. Critically, the Spearman correlation of microbial abundance prediction on the experimental validation set showed a consistent improvement over the fine-tuning epochs, reaching 0.85. This highlights the model’s ability to effectively transfer knowledge from simulated environments and adapt to experimental data complexities.

### Development and optimization

The optimal configuration was obtained using Bayesian hyperparameter optimization, with the primary objective of maximizing the Spearman rank correlation for microbial abundance prediction. This process was carries out by tuning hyperparameters, including the model’s hidden dimension, attention head, edge dropout, feature masking, and the weight of the rank loss component in the combined loss function. The overall relationship between the hyperparameters and the achieved Spearman correlation is shown in Supplementary Fig. 6. Specifically, varying the feature masking revealed an optimal range around 0.1, where higher masking led to a slight decline in performance. Similarly, an edge dropout centered around 0.2 was found to be most effective for microbial abundance prediction. The influence of the rank loss weight showed stable performance around zero.

Further investigation into the interplay between hidden dimension sizes (D256, D512, D768, and D1024) and attention heads (H8, H12, and H16) was performed, and SIMBA-D768-H12 outperformed others with a Spearman correlation above 0.8 (see Supplementary Fig. 7). Across all models, Spearman scores generally showed a trend of stabilizing after approximately 20 epochs. The complete set of optimized hyperparameters used in SIMBA is provided in Table 1.

**Table 1:** Final optimized hyperparameter configurations for SIMBA

Hyperparameter	Hidden dim.	Attn. head	Edge dropout	Feature mask	Tweedie power	Ranking loss weight
Optimized value	768	12	0.1	0.1	1.5	0

Values were determined through Bayesian hyperparameter optimization targeting Spearman correlation for microbial abundance.

A critical aspect of modeling microbial abundances is the choice of an appropriate loss function, given the characteristic zero-inflation and skewed distribution of such data. We systematically evaluated several loss functions for the abundance prediction task during the fine-tuning stage. From Supplementary Fig. 8, which represents the Spearman correlation scores with different loss functions, the Tweedie loss with a power parameter of 1.5 demonstrated superior performance in capturing the abundance distribution, outperforming both the KL divergence and the Huber loss functions.

### Microbial and metabolite prediction insights

**Fig. 3:**
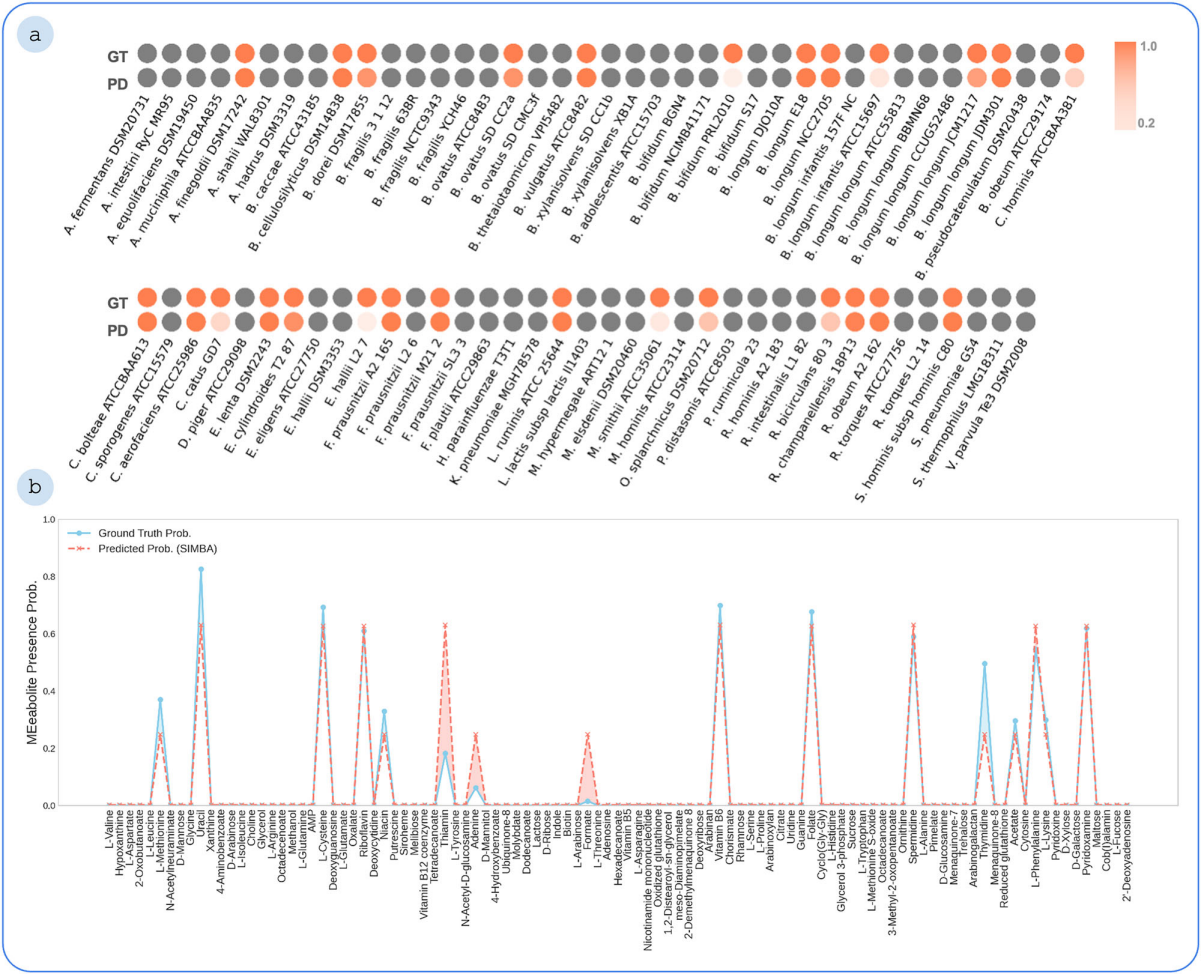
Comparison of ground truth and predictions for an exemplary sample. **a** SIMBA performance in predicting microbial presence and abundance. Each pair of circles represents a microbe: the top (GT) shows ground truth, and the bottom (PD) shows model prediction. Gray circles indicate absence, while colored circles indicate presence. The color intensity reflects how well the predicted abundance matches the ground truth, computed as a ratio between predicted and actual values. A higher intensity suggests a closer match. **b** Comparison of ground truth probabilities and predicted probabilities for different metabolites. (GD ground truth, PD prediction).

At the individual sample level, Fig. 3 provides a detailed comparison between the ground truth data and model predictions for a representative experimental sample. The model performs well in predicting both the presence of all microbes in the community and captures the qualitative landscape of metabolites’ cross-feeding interactions. Absence is indicated by gray circles, while colored circles denote presence. The color intensity of prediction circles reflects the concordance with ground truth abundance, computed as a ratio, where a higher intensity indicates a closer match. This example is representative of the general predictive trends observed across validation samples, showing the model’s consistent ability to capture microbial presence and relative abundance patterns.

While the model excels at predicting the presence or absence of species, the figure also reveals quantitative discrepancies in the predicted relative abundances. For instance, the abundances for key species, such as *B. longum infantis* ATCC 15697 and *M. smithii* ATCC 35061, are under-predicted relative to the ground truth (indicated by a paler orange circle for the prediction). These quantitative differences, which are the primary contributors to the model’s strong Spearman correlation of 0.85, can stem from several factors. The underlying metabolic networks may not perfectly capture the metabolic efficiency of each microbe, or complex synergistic interactions not fully represented in our pairwise simulations could amplify the growth of certain species in vivo.

A similar pattern is observed for metabolite presence probabilities. The model is highly accurate in identifying which metabolites are likely to be cross-fed (with an accuracy of 0.96 and an F1-score of 0.83). However, it sometimes over- or under-predicts the precise probability of these exchanges. This suggests that while the model correctly captures the potential for metabolic handoffs, predicting their exact frequency may require additional information, such as regulatory constraints. These observations are valuable, as they demonstrate the model’s strength in capturing the qualitative ecological landscape while pointing towards specific areas, like model parameterization and higher-order interaction modeling, for future quantitative improvements.

## Discussion

Modeling the human gut microbiome is particularly challenging due to the heterogeneous, zero-inflated, and compositionally biased nature of abundance data. Community structure reflects not only taxonomic presence but also metabolic activity and ecological interactions, including cross-feeding, competition, and syntrophy. Approaches that rely solely on abundance data or simplified interaction assumptions often miss this underlying microbiological and ecological complexity.

To address these challenges, we integrated experimental data with mechanistic modeling and graph-based learning to form a multimodal representation of microbial communities. Using GEMs, we simulated 2850 pairwise interactions to map a rich landscape of potential metabolic cross-feeding-an ecological signal often inaccessible through experimental settings alone. These simulations seed a heterogeneous interaction graph that incorporates functional embeddings, pathway annotations, and probabilistic metabolite cross-feeding data.

Building on this representation, we developed a simulation-augmented GNN framework tailored to the microbiome domain. Unlike baselines, which struggled to exceed a Spearman correlation of 0.6, our architecture introduces a heterogeneous graph transformer with edge attributes embedded directly into the attention mechanism. This enhancement enables biologically grounded modeling of both the strength and directionality of simulated microbial interactions. The training pipeline mirrors this design: a three-stage strategy-self-supervised representation learning, supervised pretraining on simulated data, and fine-tuning on experimental abundance profiles-facilitated transfer from in silico simulations to in vivo observations, stabilized optimization, and yielded a Spearman correlation of 0.85 on experimental validation data. Bayesian hyperparameter optimization selected moderately large hidden dimensions (768), 12 attention heads, and low edge dropout and feature-masking rates (both set to 0.1). A Tweedie loss (power = 1.5) was particularly effective for zero-inflated, right-skewed abundance distributions, outperforming alternatives like KL divergence and Huber loss.

Beyond predictive performance, SIMBA is designed to serve as a foundation for microbiological interpretability. The explicit inclusion of microbe, metabolite, and pathway nodes in our heterogeneous graph sets a structure where model internals, such as attention weights, could, in principle, correspond to a mechanistic hypothesis. To make such signals accessible to domain experts, we envision pairing predictive modeling with natural language querying over knowledge graphs. Our parallel effort, KODA, is an LLM-powered framework that translates natural language questions into queries over a complex microbiome knowledge graph^23^. A combined workflow in which SIMBA learns predictive dependencies and a KODA-like interface supports conversational interrogation offers a path toward actionable, interpretable insight.

These results must be interpreted in light of data characterization. A key feature of sequencing-derived microbial abundance data is its compositional nature: each sample is a vector of parts that sum to a constant. As a result, only ratios among taxa are intrinsically meaningful. Within a single sample, discussing relative dominance is valid, but cross-sample comparisons of raw counts or proportions are susceptible to compositional biases unless appropriate transformations e.g., log-ratio methods, are used^24–26^. By contrast, in untargeted LC/GC-MS metabolomics, the raw signal intensity of a feature is not a direct measure of absolute concentration because response depends on compound-specific ionization efficiency and matrix effects. Therefore, comparing the intensity of two different metabolites within the same sample is not interpretable without compound-specific calibration (ideally using isotopically labeled internal standards). The standard practice is relative quantification across samples for the same metabolite, e.g., fold-changes between groups, or targeted absolute quantification with appropriate standards^27–29^. This asymmetry has implications for multi-omics integration: microbiome features are best analyzed through ratios (or balances), while untargeted metabolomics typically supports between-sample comparisons for a given metabolite rather than within-sample comparisons across different metabolites. Accordingly, metabolomics-based validation of SIMBA’s hypotheses should emphasize between-condition differences for the same metabolite, not absolute within-sample comparisons across metabolites^25,30^.

By jointly modeling species presence, metabolic capacities, and cross-feeding patterns, our approach highlights keystone taxa and metabolic bottlenecks. This could offer a mechanistic foundation for developing targeted therapeutic or dietary interventions.

This initial validation was performed in a well-characterized cohort under a high-fiber diet. While this setting supports careful control, it constrains external validity. SIMBA relies on context-specific, diet-constrained metabolic simulations to generate interaction graphs, and the current model was trained on graphs tailored to the microbes and dietary constraints of this dataset. Applying the framework to cohorts with differing community or dietary interventions will require de novo generation of simulation-based graphs, which was beyond the scope of the present study.

A natural next step is to apply the pipeline across diverse cohorts and diets. For each cohort, we will generate the corresponding diet-constrained simulations, enabling a principled assessment of out-of-distribution generalization for the GNN and a mechanistic view of how diet reshapes interaction networks and community structure. Such comparative studies will be invaluable for moving towards truly personalized microbiome modeling.

Our implementation currently relies on simulated pairwise interactions and on the fidelity of genome-scale reconstructions and the flux-sampling scheme, which may not fully capture the constraints of in vivo environments. The present graph structure is also static and does not explicitly account for temporal dynamics or spatial organization, both of which are critical for shaping microbial behavior. Future extensions could incorporate longitudinal datasets and host-microbiome interactions, and could refine predictions using multi-omics integration.

As the framework scales to larger communities and higher-dimensional inputs, interpretability and computational efficiency will become increasingly important. Explainable GNN techniques and contrastive graph distillation could offer actionable insights while preserving model transparency. Complementary strategies, such as amortized or surrogate simulators to accelerate graph generation^31,32^, uncertainty quantification to convey confidence^33^, and standardized reporting^34^, can further support robust, generalizable applications without compromising the mechanistic fidelity central to SIMBA.

Accurate prediction of gut microbial community assembly requires modeling tools that capture the mechanistic basis of interspecies interactions rather than relying solely on statistical associations. SIMBA is an initial effort that encodes metabolic dependencies directly into the architecture of graph learning and generates predictions that are mechanistically grounded. This synthesis of simulation and deep learning transforms static abundance tables into living ecological networks, which enables the discovery of keystone taxa, metabolic bottlenecks, and potential intervention targets. While developed under a specific dietary context, the framework is inherently adaptable to new diets, cohorts, and perturbation scenarios. As such, SIMBA provides a generalizable and scalable approach for microbiome modeling, offering predictive accuracy, mechanistic insight, and flexibility to support hypothesis generation and guide targeted interventions.

## Methods

### Microbial abundance data and selection of GEMs

Microbial abundance profiles were obtained from 186 individuals participating in a high-fiber dietary intervention study^35^. These profiles served as the target variable for our GNN model. All identifiable microbial taxa from the cohort’s microbiome profiles were extracted. For each taxon, corresponding GEMs were retrieved from the AGORA database^36^. This resulted in 76 unique GEMs representing key members of the cohort’s gut microbiome (see Supplementary Table 1 for the list of microbes involved in the simulations). Each GEM encodes a stoichiometric reconstruction of its organism’s metabolic network, which enables the simulation of metabolic fluxes^37^.

### Simulation of pairwise microbial interactions

From each simulation, we extracted cross-fed metabolites, corresponding biomass fluxes, and metabolic pathway activity scores for each microbe. Simulations were conducted using constraint-based flux sampling, a Monte Carlo-based method that explores the feasible metabolic states of the paired microbes under a shared, high-fiber dietary environment and enables statistical quantification of potential interactions. Details of procedures are described in the following subsections.

#### Model merging and shared environment

For each microbial pair, the corresponding GEMs were combined into a multi-compartment model^38^. Metabolites and reactions from each organism were uniquely labeled to avoid conflicts, and a shared pool compartment was introduced to facilitate metabolite exchange. Overlapping exchange reactions from both models were linked *via* pool metabolites, with bidirectional transport reactions allowing secretion or uptake between microbes.

#### Dietary constraints

For all simulations, GEMs were constrained to mimic a high-fiber diet. An averaged high-fiber diet profile, based on experimental data^35^, was used to define the input metabolic fluxes into the pool compartment (Supplementary Table 2). All simulations were performed under anaerobic conditions to reflect the dominant environment of the human colon. Non-dietary exchange reactions were initially closed to uptake, allowing only secretion into the shared compartment unless a metabolite was part of the defined medium. Metabolic byproducts were allowed to exit the system to simulate environmental turnover, with upper bounds set to 1000.

#### Flux sampling and objective function

For each pairwise simulation, the objective was set to maximize the joint biomass production of the pair. To ensure both microbes could grow, each individual biomass reaction was constrained to be at least 10% of its monoculture optimal growth rate, as determined by flux balance analysis^39^, under the same dietary conditions. Flux sampling was then performed using the *optgp* method^40^, generating 10,000 flux distributions with a thinning factor of 100^41^. Convergence of flux samples was assessed using the Geweke diagnostic test^42^.

#### Identification of cross-feeding metabolites

Metabolites were classified as cross-fed if secreted by one microbe and concurrently taken up by the other via the pool compartment. To quantify this interaction, Spearman correlation analysis was performed on the fluxes of the paired microbes for each shared metabolite: a strong negative correlation (e.g., threshold < −0.5) indicated potential cross-feeding. Directionality, frequency of exchange events, and probabilities of metabolite presence (proportion of events observed across 10,000 samples) were recorded as edge features. Only metabolites with exchange fluxes exceeding a certain threshold and absent from the initial dietary input were retained.

#### Pathway activity scoring

Pathway-level activity was quantified for each microbe from flux distributions. Reactions were mapped to metabolic pathways using annotations from the virtual metabolic human (VMH) and KEGG databases^43,44^. These pathway fingerprints, calculated by aggregating the fluxes of constituent reactions, were used to compare metabolic activity profiles across microbial interactions (Supplementary Fig. 1).

To investigate the functional relationships among microbial strains, we performed hierarchical agglomerative clustering based on their pathway activity scores. A standardized matrix of strains (rows) by metabolic pathways (columns) was generated, with absent pathways assigned a value of zero. Scores were scaled to zero mean and unit variance per pathway before hierarchical agglomerative clustering using Ward’s variance minimization algorithm and Euclidean distance^45^. This algorithm iteratively merges clusters in a manner that minimizes the total within-cluster variance at each step. Results were visualized as a dendrogram showing metabolic similarity (Supplementary Fig. 2).

### Implementation details of SIMBA

Our heterogeneous graphs consisted of three node types-microbes, metabolites, and pathways, and multiple biologically meaningful edge types. Microbe-metabolite edges captured directional interactions based on metabolite probabilities inferred from simulations, effectively modeling ecological interactions such as cross-feeding. Microbe-pathway edges represent functional associations, linking microbes to pathways with nonzero activity in simulations. Additionally, microbe-microbe edges were added based on pathway profile similarity, computed using cosine similarity between log-transformed pathway activity vectors. These similarity-based edges were introduced to capture potential ecological relationships, such as competition or functional redundancy, that are not represented by the direct cross-feeding interactions from our simulations. To reduce spurious connections and maintain graph sparsity, only edges with similarity scores above 0.85 were retained (Fig. 2b). This allowed the GNN to directly pass messages between functionally similar microbes, enriching its representation of the overall community structure.

#### Node and edge features

Node features were designed to provide the model with a multi-faceted representation of each biological entity.

Microbe nodes were characterized using concatenated feature vectors that integrate information from proteome, metabolome, and pathway activity levels:

- Protein-level embeddings, generated *via* the ESM-2 model^46^, yielding 2560-dimensional vectors from the NCBI database^47^, averaged across all proteins in each genome^48^. This enables the model to capture relationships based on fundamental biological capabilities that may not be fully reflected in curated metabolic pathway definitions alone.
- Pathway fingerprints log-transformed (log1p) to mitigate skewness, representing the metabolic activity profile of each microbe, derived from pairwise simulations.
- Metabolite fingerprints, representing probabilities of metabolite production/consumption estimated from flux sampling in our simulations.

To allow the model to learn their roles within the community, pathway and metabolite nodes were initialized with randomly learnable embeddings of dimensions 256 and 128, respectively. These embeddings are optimized during the training process to best represent the functions of each pathway and metabolite in the context of the microbial ecosystem.

#### Enhanced heterogeneous graph transformer architecture

We extended the standard HGT architecture^49^ by integrating scalar edge attributes directly into the attention mechanism. This enhancement enabled the model to weigh neighbor contributions based not only on node features but also on interaction strengths, as informed by metabolite fluxes and microbial abundances. Our resulting model, (SIMBA), comprises three transformer layers, each with 768-dimensional hidden states and 12 attention heads-hyperparameters optimized through grid search. To improve training stability and mitigate overfitting, we applied residual connections, layer normalization, and a dropout rate of 0.2.

Task-specific output heads were designed to support multiple prediction objectives:

- Microbial presence was predicted using a sigmoid-activated binary classifier (auxiliary).
- Microbial abundances were estimated using a softmax layer: the ground truth relative abundances for each sample are normalized to sum to 1, and the softmax produces a distribution over species accordingly (primary).
- Metabolite fingerprints were predicted as logits over binary indicators of metabolite production capability (used in pretraining).

The presence head outputs binary labels indicating whether a microbe is expected to occur in a given sample, while the abundance and metabolite heads yield normalized probabilities for microbial abundances and metabolite presence in experimental or simulated communities.

#### Model input for prediction

Each prediction made by the model corresponds to one individual microbiome sample. All samples share a common mechanistic scaffold: the global heterogeneous graph that connects microbes, metabolites, and pathways derived from simulations. What distinguishes one sample from another is not the graph topology but the membership of microbes actually detected in that individual and their corresponding abundance values.

For each sample, the model receives this shared graph together with a non-learned membership mask that identifies the observed taxa. The model then predicts which microbes should be present and their relative abundances within the community. In this way, the network leverages a fixed, biologically informed interaction map while adapting its output to the sample-specific microbial profile.

A non-learned membership mask is applied inside the abundance head to define which taxa have observed abundances in that sample and to restrict the softmax/loss to that subset. The encoder and the presence head do not receive the mask or any ground-truth presence vector as input features. Thus, presence prediction is not trivialized by label leakage, while abundance prediction remains the primary endpoint.

In essence, the model performs node-level regression on the shared scaffold with sample-specific normalization at the output layer. Through its transformer layers, the GNN updates microbe representations based on their features and their position within the interaction network. The final heads then use these updated representations to predict each microbe’s presence and relative abundance. The prediction of metabolite presence was an auxiliary task used during pretraining, with the primary goal of the fine-tuned model being the prediction of microbial abundances.

#### Training strategy and loss functions

We structured our training pipeline into three distinct stages to enhance learning efficacy:

Self-supervised learning (SSL): In the initial stage, we employed graph contrastive learning (GraphCL)^50^ with a temperature parameter *τ* = 0.10 and a large batch of 256 negatives to learn task-agnostic node embeddings.

Supervised pretraining: In this stage, we used simulation graphs to train the model on microbial abundances and metabolite presence (metabolite cross-feeding probability) predictions. Microbial abundances were optimized using the Tweedie loss with a power parameter of 1.5, selected based on performance comparisons with power values of 1.1, 1.5, and 1.8. The Tweedie loss is defined as^51^:

1 $${{\mathcal{L}}}_{{\rm{Tw}}}(\,y,\hat{y};p)=2\left[\frac{{y}^{2-p}}{(1-p)(2-p)}-\frac{y{\hat{y}}^{1-p}}{1-p}+\frac{{\hat{y}}^{2-p}}{2-p}\right],$$

where *y* denotes the true abundance, $\hat{y}$ is the predicted abundance, and *p* is the power parameter (*p* = 1.5). Metabolite presence was optimized with binary cross-entropy (BCE) during supervised pretraining^52^.

Fine-tuning: While our model supports joint training on microbial and metabolite outputs, the BCE loss term for cross-feeding metabolites was disabled (*α*_flux_ = 0), as metabolite-level labels were unavailable for this dataset. We retained the BCE loss for microbial presence prediction (auxiliary) and the Tweedie loss for abundance regression (primary). To avoid label leakage, the membership mask is applied only inside the abundance head’s softmax and loss and is not provided to the encoder or the presence head. Additionally, we evaluated a pairwise hinge ranking loss (margin 0.1) with weighting factors *α*_rank_ ∈ {0.05, 0.10, 0.20}. However, using the ranking loss did not yield any improvements in the validation Spearman correlation. As a result, the final model was trained with *α*_rank_ = 0.

Feature masking and edge dropout of 0.1 were applied at all training stages.

The stage-specific total loss is (Table 2):

2 $${{\mathcal{L}}}_{{\rm{total}}}={\alpha }_{{\rm{con}}}{{\mathcal{L}}}_{{\rm{CL}}}+{\alpha }_{{\rm{pres}}}{{\mathcal{L}}}_{{\rm{BCE}}}+{\alpha }_{{\rm{abund}}}{{\mathcal{L}}}_{{\rm{Tw}}}+{\alpha }_{{\rm{flux}}}{{\mathcal{L}}}_{\,\text{BCE}}^{\text{met}\,}$$

**Table 2:** Loss-weight schedule used in the final model

Stage	*α*_con_	*α*_pres_	*α*_abund_	*α*_flux_
Self-supervised (SSL)	1	0	0	0
Supervised pretraining	–	1	1	1
Fine-tuning	–	1	1	0

Evaluation: A tenfold cross-validation scheme was used in each training phase. The dataset, containing both experimental and simulation data points, was randomly partitioned into ten equal subsets; in each iteration, one subset was used for validation and the remaining nine for training. This process was repeated until each subset had served as the validation set once, and performance metrics were averaged across folds. Unless noted otherwise, model selection prioritizes the abundance objective. The results, including Fig. 3, present averaged values to ensure representativeness and avoid bias toward training samples.

#### Baseline GNN models

To evaluate the performance of our proposed architecture, we benchmarked it against several established GNN models. We selected a set of general-purpose GNNs to represent the state-of-the-art in graph-based learning. These include GraphSAGE^53^, a widely-used inductive framework that learns by sampling and aggregating features from a node’s local neighborhood, and other representative message-passing architectures like message passing graph neural network (MPGNN)^54^ and structured world models (SWM-GNN)^55^, as well as GNN^56^. The key distinction of these baselines is their sole reliance on microbial node features and metabolite production fluxes, without explicit edge-value integration. Due to their tendency to overfit, these baseline models were maintained smaller than the heterogeneous model.

#### Hyperparameter optimization and evaluation metrics

Bayesian hyperparameter optimization was used to identify optimal model configurations. For evaluation, Spearman’s rank correlation served as our primary metric to capture the relative ordering of microbial abundances, which is an essential aspect of ecological validity. The final model configuration included a hidden dimension of 768, a dropout rate of 0.2, an edge dropout of 0.1, a feature masking rate of 0.1, and 12 attention heads.

## Supplementary information

Supplementary information

## Data Availability

SIMBA is implemented in Python and available from GitHub (https://github.com/mofradlab/simba). The pretrained model checkpoints generated in this study are deposited on Zenodo at (https://zenodo.org/records/15521581). All genome-scale metabolic models used were obtained from the AGORA database. Processed datasets and scripts necessary to reproduce the analyses presented in this manuscript are provided within the GitHub repository. Additional raw data supporting the findings of this study are available from the corresponding author upon reasonable request.

## Code availability

SIMBA is implemented in Python and available from GitHub (https://github.com/mofradlab/simba).
