# Supplementary material for: SIMBA-GNN: mechanistic graph learning for microbiome prediction
Source: NPJ Syst Biol Appl. 2025 Dec 12;12:8. doi: 10.1038/s41540-025-00631-w (PMC12796443; doi:10.1038/s41540-025-00631-w)
Supplement: Supplementary file 1 — Supplementary information [file 41540_2025_631_MOESM1_ESM.pdf]

SIMBA-GNN: Mechanistic Graph Learning for Microbiome Prediction

Javad Aminian-Dehkordi*[1], Mohammad Parsa*[1], Andrew Dickson[1], and Mohammad R.K. Mofrad†[1,2]

[1]Molecular Cell Biomechanics Laboratory, Departments of Bioengineering and Mechanical Engineering, University of California, Berkeley, California

[2]Molecular Biophysics and Integrative Bioimaging Division, Lawrence Berkeley National Laboratory, Berkeley, California

*Equal contribution

†Corresponding author: `mofrad@berkeley.edu`

1 Supplementary information

Supplementary Table 1: List of strains used to study pairwise simulations grouped by Phylum.

Phylum	Species	Strain	Gram stain
Actinomycetota	*Adlercreutzia equolifaciens*	DSM 19450	Gram-positive
	Bifidobacterium adolescentis	ATCC 15703	Gram-positive
	Bifidobacterium bifidum	BGN4	Gram-positive
	Bifidobacterium bifidum	NCIMB 41171	Gram-positive
	Bifidobacterium bifidum	PRL2010	Gram-positive
	Bifidobacterium bifidum	S17	Gram-positive
	Bifidobacterium longum	DJO10A	Gram-positive
	Bifidobacterium longum	E18	Gram-positive
	Bifidobacterium longum	NCC2705	Gram-positive
	Bifidobacterium longum	157F NC	Gram-positive
	Bifidobacterium longum	ATCC 15697	Gram-positive
	Bifidobacterium longum	ATCC 55813	Gram-positive
	Bifidobacterium longum	BBMN68	Gram-positive
	Bifidobacterium longum	CCUG 52486	Gram-positive
	Bifidobacterium longum	JCM 1217	Gram-positive
	Bifidobacterium longum	JDM301	Gram-positive
	Bifidobacterium pseudocatenulatum	DSM 20438	Gram-positive
	Collinsella aerofaciens	ATCC 25986	Gram-positive
	Eggerthella lenta	DSM 2243	Gram-positive
Bacteroidota	*Alistipes finegoldii*	DSM 17242	Gram-negative
	Alistipes shahii	WAL 8301	Gram-negative
	Bacteroides caccae	ATCC 43185	Gram-negative
	Bacteroides cellulosilyticus	DSM 14838	Gram-negative
	Bacteroides dorei	DSM 17855	Gram-negative
	Bacteroides fragilis	3 1 12	Gram-negative

Continued on next page

Supplementary Table 1 – Continued from previous page.

Phylum	Species	Strain	Gram stain
	Bacteroides fragilis	638R	Gram-negative
	Bacteroides fragilis	NCTC 9343	Gram-negative
	Bacteroides fragilis	YCH46	Gram-negative
	Bacteroides ovatus	ATCC 8483	Gram-negative
	Bacteroides ovatus	SD CMC 3f	Gram-negative
	Bacteroides ovatus	SD CC 2a	Gram-negative
Bacteroidota	*Bacteroides thetaiotaomicron*	VPI 5482	Gram-negative
	Bacteroides vulgatus	ATCC 8482	Gram-negative
	Bacteroides xylanisolvens	SD CC 1b	Gram-negative
	Bacteroides xylanisolvens	XB1A	Gram-negative
	Odoribacter splanchnicus	DSM 20712	Gram-negative
	Parabacteroides distasonis	ATCC 8503	Gram-negative
	Acidaminococcus fermentans	DSM 20731	Gram-negative
	Acidaminococcus intestini	RyC MR95	Gram-negative
	Anaerostipes hadrus	DSM 3319	Gram-positive
	Blautia obeum	ATCC 29174	Gram-positive
	Clostridium bolteae	ATCC BAA 613	Gram-positive
	Clostridium sporogenes	ATCC 15579	Gram-positive
	Coprococcus catus	GD 7	Gram-positive
Firmicutes	*Eubacterium cylindroides*	T2 87	Gram-positive
	Eubacterium eligens	ATCC 27750	Gram-positive
	Eubacterium hallii	DSM 3353	Gram-positive
	Eubacterium hallii	L2 7	Gram-positive
	Faecalibacterium prausnitzii	A2 165	Gram-positive
	Faecalibacterium prausnitzii	L2 6	Gram-positive
	Faecalibacterium prausnitzii	M21 2	Gram-positive
	Faecalibacterium prausnitzii	SL3 3	Gram-positive

Continued on next page

Supplementary Table 1 – Continued from previous page.

**Phylum**	**Species**	**Strain**	**Gram stain**
Firmicutes	*Flavonifractor plautii*	ATCC 29863	Gram-positive
	Lactobacillus ruminis	ATCC 25644	Gram-positive
	Lactococcus lactis	Il1403	Gram-positive
	Megamonas hypermegale	ART12 1	Gram-negative
	Megasphaera elsdenii	DSM 20460	Gram-negative
	Prevotella ruminicola	23	Gram-negative
	Roseburia hominis	A2 183	Gram-positive
	Roseburia intestinalis	L1 82	Gram-positive
	Ruminococcus bicirculans	80 3	Gram-positive
	Ruminococcus champanellensis	18P13	Gram-positive
	Ruminococcus obeum	A2 162	Gram-positive
	Ruminococcus torques	ATCC 27756	Gram-positive
	Ruminococcus torques	L2 14	Gram-positive
	Staphylococcus hominis	C80	Gram-positive
	Streptococcus pneumoniae	G54	Gram-positive
	Streptococcus thermophilus	LMG 18311	Gram-positive
	Veillonella parvula	DSM 2008	Gram-negative
Pseudomonadota	*Campylobacter hominis*	ATCC BAA 381	Gram-negative
	Desulfovibrio piger	ATCC 29098	Gram-negative
	Haemophilus parainfluenzae	T3T1	Gram-negative
	Klebsiella pneumoniae	MGH78578	Gram-negative
Tenericutes	*Mycoplasma hominis*	ATCC 23114	Lacks cell wall
Verrucomicrobiota	*Akkermansia muciniphila*	ATCC BAA 835	Gram-negative
Euryarchaeota	*Methanobrevibacter smithii*	ATCC 35061	Gram-positive

Supplementary Table 2: Constraints applied to each GEM prior to the main simulations representing the high-fiber diet.

**Category**	**Metabolite Name**	**Exchange Reaction ID**	**Dietary Flux** $(\frac{\text{mmol}}{\text{g}_{\text{DW}}\cdot\text{h}})$
Carbohydrates	L-arabinose	`EX_arab_L(e)`	0.047
	Cellobiose	`EX_cellb(e)`	0.019
	2-deoxy-D-ribose	`EX_drib(e)`	0.047
	D-Fructose	`EX_fru(e)`	0.039
	L-fucose	`EX_fuc_L(e)`	0.039
	D-Galactose	`EX_gal(e)`	0.039
	D-glucose	`EX_glc(e)`	0.039
	Lactose	`EX_lcts(e)`	0.019
	Maltose	`EX_malt(e)`	0.019
	D-Mannose	`EX_man(e)`	0.039
	Melibiose	`EX_melib(e)`	0.019
	D-ribose	`EX_rib_D(e)`	0.047
	L-Rhamnose	`EX_rmn(e)`	0.039
	Sucrose	`EX_sucr(e)`	0.019
	Trehalose	`EX_tre(e)`	0.019
	D-xylose	`EX_xyl_D(e)`	0.047
	Starch	`EX_strch1(e)`	0.068
	Amylopectin	`EX_amylopect900(e)`	3.4×10^{-4}
	Amylose	`EX_amylose300(e)`	0.001
	Arabinan	`EX_arabinan101(e)`	0.003
	Larch arabinogalactan	`EX_arabinogal(e)`	4.8×10^{-4}
	Arabinoxylan	`EX_arabinoxyl(e)`	0.007
	Beta-glucan	`EX_bglc(e)`	1.5×10^{-6}
	Cellulose	`EX_cellul(e)`	0.001
	Dextran 40, 1,6-alpha-D-Glucan	`EX_dextran40(e)`	0.004

Continued on next page

Category	Metabolite Name	Exchange Rxn ID	Dietary Flux Value
Carbohydrates	Carob galactomannan	EX_galmannan(e)	3.1×10^{-4}
	Konjac glucomannan	EX_glcmannan(e)	0.001
	Homogalacturonan	EX_homogal(e)	0.003
	Chicory inulin	EX_inulin(e)	0.010
	Kestopentaose	EX_kestopt(e)	0.062
	Levan, 1000 fructose units	EX_levan1000(e)	3.1×10^{-4}
	LicheninfromIcelandic moss	EX_lichn(e)	0.002
	Laminarin	EX_lmn30(e)	0.010
	Pectin	EX_pect(e)	0.001
	Pullulan	EX_pullulan1200(e)	2.6×10^{-4}
	Raffinose	EX_raffin(e)	0.104
	Potato rhamnogalacturonan I	EX_rhamnogalurI(e)	0.0003
	Wine rhamnogalacturonan II	EX_rhamnogalurII(e)	0.006
	Resistant starch	EX_starch1200(e)	0.0002
	Oat spelt xylan	EX_xylan(e)	0.0007
	Xyluglucan	EX_xyluglc(e)	0.0003
	N-acetyl-D-glucosamine	EX_acgam(e)	1
	N-acetyl-D-mannosamine	EX_acmana(e)	1
	N-acetylneuraminate	EX_acnam(e)	1
Lipids & Fatty Acids	Arachidonate	EX_arachd(e)	0.002
	Cholesterol	EX_chsterol(e)	0.002
	Glycerol	EX_glyc(e)	0.899
	Hexadecanoate (n-C16:0)	EX_hdca(e)	0.198
	Hexadecenoate (n-C16:1)	EX_hdcea(e)	0.018
	Linoleate	EX_lnlc(e)	0.179 554 61
	Alpha-linolenate	EX_lnlnca(e)	0.008

Continued on next page

Category	Metabolite Name	Exchange Rxn ID	Dietary Flux Value
Lipids & Fatty Acids	Gamma-linolenate	EX_lnlncg(e)	0.008
	Octadecanoate (n-C18:0)	EX_ocdca(e)	0.084
	Octadecenoate (n-C18:1)	EX_ocdcea(e)	0.341
	Octanoate (n-C8:0)	EX_octa(e)	0.006
	Tetradecanoate (n-C14:0)	EX_ttdca(e)	0.034
	1,2-Diacyl-sn-glycerol (dioctadecanoyl)	EX_12dgr180(e)	1
	Laurate	EX_ddca(e)	1
	lanosterol	EX_lanost(e)	1
Amino Acids	L-alanine	EX_ala_L(e)	1
	L-arginine	EX_arg_L(e)	0.15
	L-asparagine	EX_asn_L(e)	0.225
	L-aspartate	EX_asp_L(e)	0.225
	L-cysteine	EX_cys_L(e)	1
	L-glutamine	EX_gln_L(e)	0.18
	L-glutamate	EX_glu_L(e)	0.18
	Glycine	EX_gly(e)	0.45
	L-histidine	EX_his_L(e)	0.15
	L-isoleucine	EX_ile_L(e)	0.15
	L-leucine	EX_leu_L(e)	0.15
	L-lysine	EX_lys_L(e)	0.15
	L-methionine	EX_met_L(e)	0.18
	L-phenylalanine	EX_phe_L(e)	1
	L-proline	EX_pro_L(e)	0.18
	L-serine	EX_ser_L(e)	1
	L-threonine	EX_thr_L(e)	0.225
	L-tryptophan	EX_trp_L(e)	0.082

Continued on next page

Category	Metabolite Name	Exchange Rxn ID	Dietary Flux Value
Amino Acids	L-tyrosine	EX_tyr_L(e)	1
	L-valine	EX_val_L(e)	0.18
	meso-2,6-Diaminoheptanedioate	EX_26dap_M(e)	1
	D-alanine	EX_ala_D(e)	1
	L-cysteinylglycine	EX_cgly(e)	1
	D-Glucosamine	EX_gam(e)	1
	D-Glutamate	EX_glu_D(e)	1
	L-Methionine Sulfoxide	EX_metsox_S_L(e)	1
	Ornithine	EX_orn(e)	1
Nucleobases & Nucleosides	Adenine	EX_ade(e)	1
	Adenosine	EX_adn(e)	1
	AMP	EX_amp(e)	1
	Cytosine	EX_csn(e)	1
	2-deoxyadenosine	EX_dad_2(e)	1
	Deoxycytidine	EX_dcyt(e)	1
	Deoxyguanosine	EX_dgsn(e)	1
	Guanine	EX_gua(e)	1
	Hypoxanthine	EX_hxan(e)	1
	Thymidine	EX_thymd(e)	1
	Uracil	EX_ura(e)	1
	Uridine	EX_uri(e)	1
	Xanthine	EX_xan(e)	1
Vitamins, Cofactors & Quinones	2-Demethylmenaquinone 8	EX_2dmmq8(e)	1
	4-Aminobenzoate	EX_4abz(e)	1
	Adenosylcobalamin	EX_adocbl(e)	1
	Biotin	EX_btn(e)	1

Continued on next page

**Category**	**Metabolite Name**	**Exchange Rxn ID**	**Dietary Flux Value**
Vitamins, Cofactors & Quinones	Cob(I)alamin	EX_cbl1(e)	1
	Folate	EX_fol(e)	1
	Menaquinone 7	EX_mqn7(e)	1
	Menaquinone 8	EX_mqn8(e)	1
	Nicotinate	EX_nac(e)	1
	Nicotinamide	EX_ncam(e)	1
	NMN	EX_nmn(e)	1
	Protoheme	EX_pheme(e)	1
	(R)-Pantothenate	EX_pnto_R(e)	1
	Pyridoxamine	EX_pydam(e)	1
	Pyridoxal	EX_pydx(e)	1
	Pyridoxal 5-phosphate	EX_pydx5p(e)	1
	Pyridoxine	EX_pydxn(e)	1
	Ubiquinone-8	EX_q8(e)	1
	Riboflavin	EX_ribflv(e)	1
	Siroheme	EX_sheme(e)	1
	Thiamin	EX_thm(e)	1
Organic Acids & Alcohols	D-gluconate	EX_glcn(e)	0.039
	D-Mannitol	EX_mnl(e)	0.039
	Oxalate(2-)	EX_oxa(e)	0.118
	2-Oxobutanoate	EX_2obut(e)	1
	3-methyl-2- oxopentanoate	EX_3mop(e)	1
	4-hydroxybenzoate	EX_4hbz(e)	1
	Acetate	EX_ac(e)	1
	Chorismate	EX_chor(e)	1
	Citrate	EX_cit(e)	1

Continued on next page

**Category**	**Metabolite Name**	**Exchange Rxn ID**	**Dietary Flux Value**
Organic Acids & Alcohols	Fe(III)dicitrate	EX_fe3dcit(e)	1
	Formaldehyde	EX_fald(e)	1
	Formate	EX_for(e)	1
	Fumarate	EX_fum(e)	1
	Glycerol 3-phosphate	EX_glyc3p(e)	1
	Indole	EX_indole(e)	1
	Methanol	EX_meoh(e)	10
	Pimelate	EX_pime(e)	1
Inorganic Ions & Gases	Calcium (Ca^{2+})	EX_ca2(e)	1
	Chloride (Cl^{-})	EX_cl(e)	1
	Cobalt (Co^{2+})	EX_cobalt2(e)	1
	Copper (Cu^{2+})	EX_cu2(e)	1
	Iron (Fe^{2+})	EX_fe2(e)	1
	Iron (Fe^{3+})	EX_fe3(e)	1
	Proton (H^{+})	EX_h(e)	1
	Hydrogen (H_2)	EX_h2(e)	1
	Water (H_2O)	EX_h2o(e)	10
	Hydrogen sulfide (H_2S)	EX_h2s(e)	1
	Potassium (K^{+})	EX_k(e)	1
	Magnesium (Mg^{2+})	EX_mg2(e)	1
	Manganese (Mn^{2+})	EX_mn2(e)	1
	Molybdate (MoO_4^{2-})	EX_mobd(e)	1
	Sodium (Na^{+})	EX_na1(e)	1
	Nitrite (NO_2^{-})	EX_no2(e)	1
	Nitrate (NO_3^{-})	EX_no3(e)	1
	Hydrogenphosphate (HPO_4^{2-})	EX_pi(e)	1

Continued on next page

**Category**	**Metabolite Name**	**Exchange Rxn ID**	**Dietary Flux Value**
Inorganic Ions & Gases	Selenate (SeO_4^{2-})	EX_sel(e)	1
	Sulfate (SO_4^{2-})	EX_so4(e)	1
	Zinc (Zn^{2+})	EX_zn2(e)	1
Miscellaneous	Choline	EX_chol(e)	1
	Oxidized glutathione	EX_gthox(e)	1
	Reduced glutathione	EX_gthrd(e)	1
	Putrescine	EX_ptrc(e)	1
	Spermidine	EX_spmd(e)	1

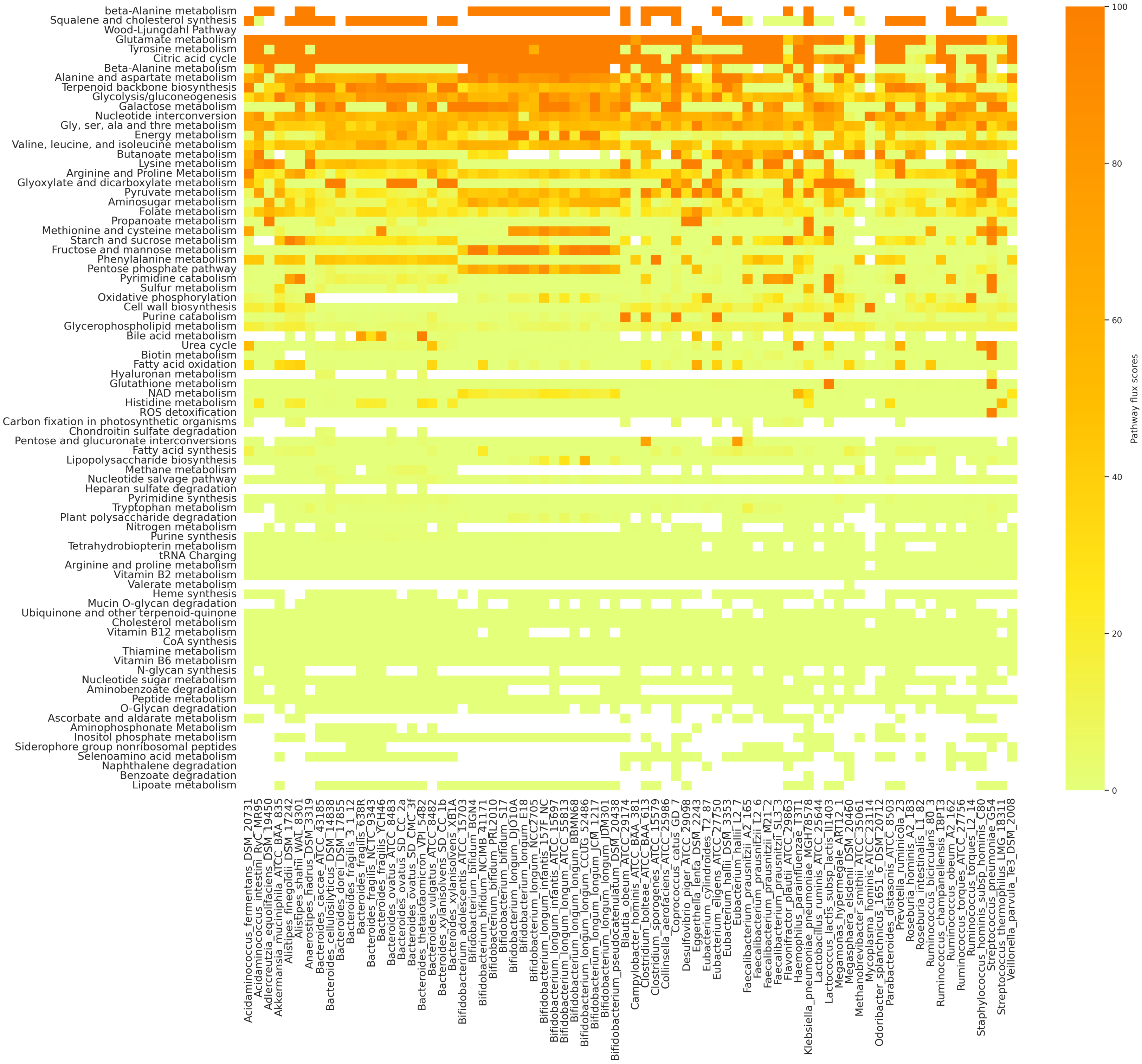

Supplementary Figure 1: Heat map representation of pathway scores for microbes involved in simulations. Rows represent metabolic pathways, columns represent microbial strains, and cell intensity indicates pathway activity scores.

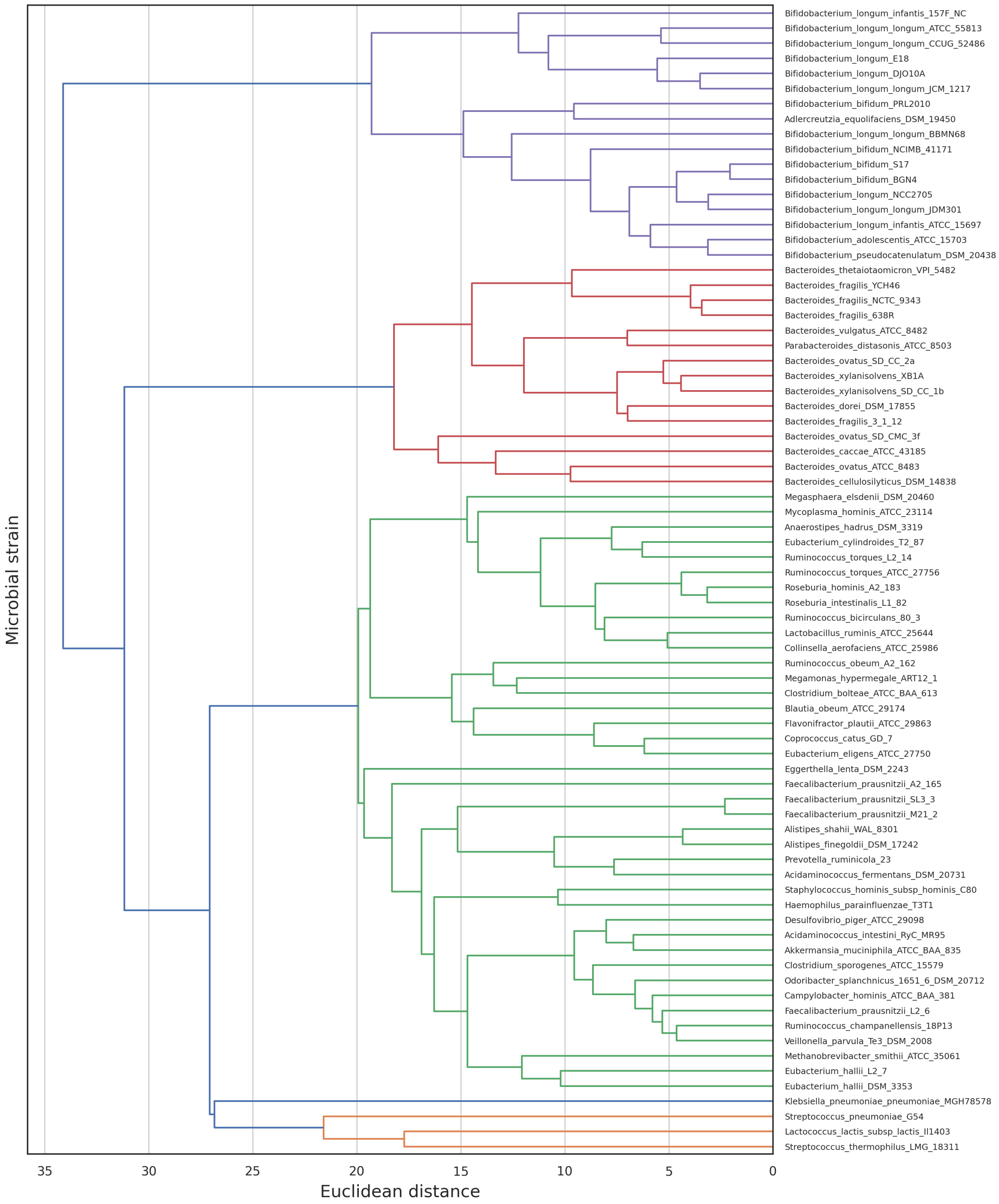

Supplementary Figure 2: Hierarchical clustering of microbial strains based on their metabolic profiles. The dendrogram was generated using Ward's linkage algorithm on standardized pathway activity scores with the Euclidean distance metric. The resulting structure groups strains with similar metabolic capabilities, where shorter horizontal branch lengths indicate a higher degree of functional similarity.

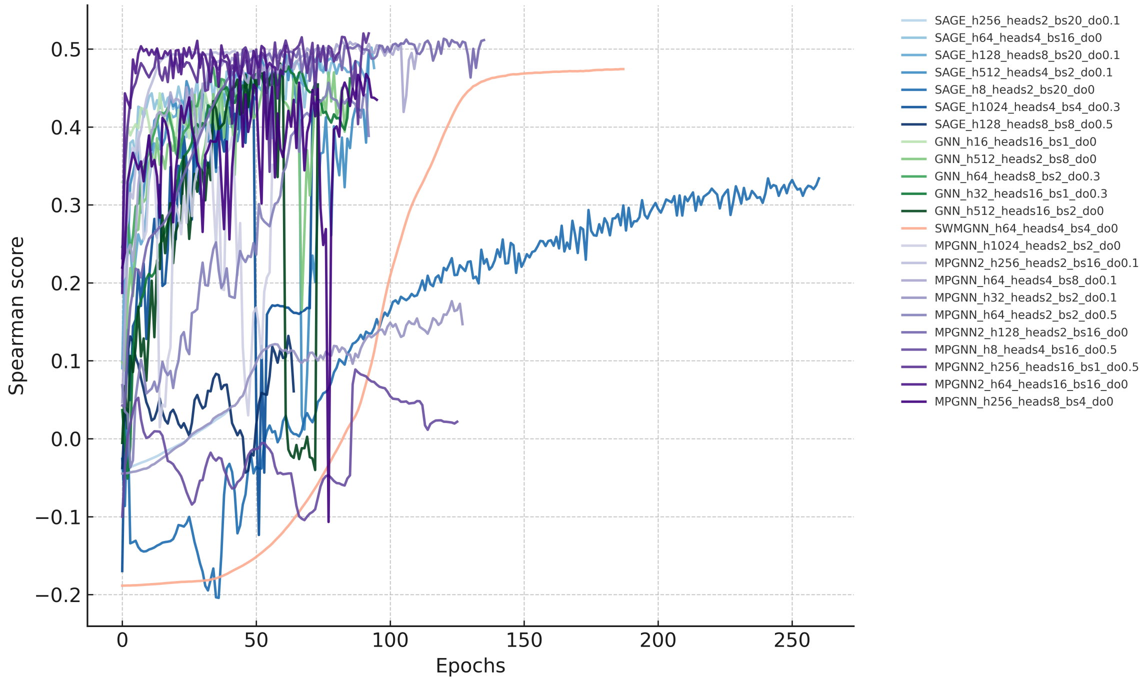

Supplementary Figure 3: Different GNN baseline model Spearman scores. The models show limited performance in microbial abundance prediction.

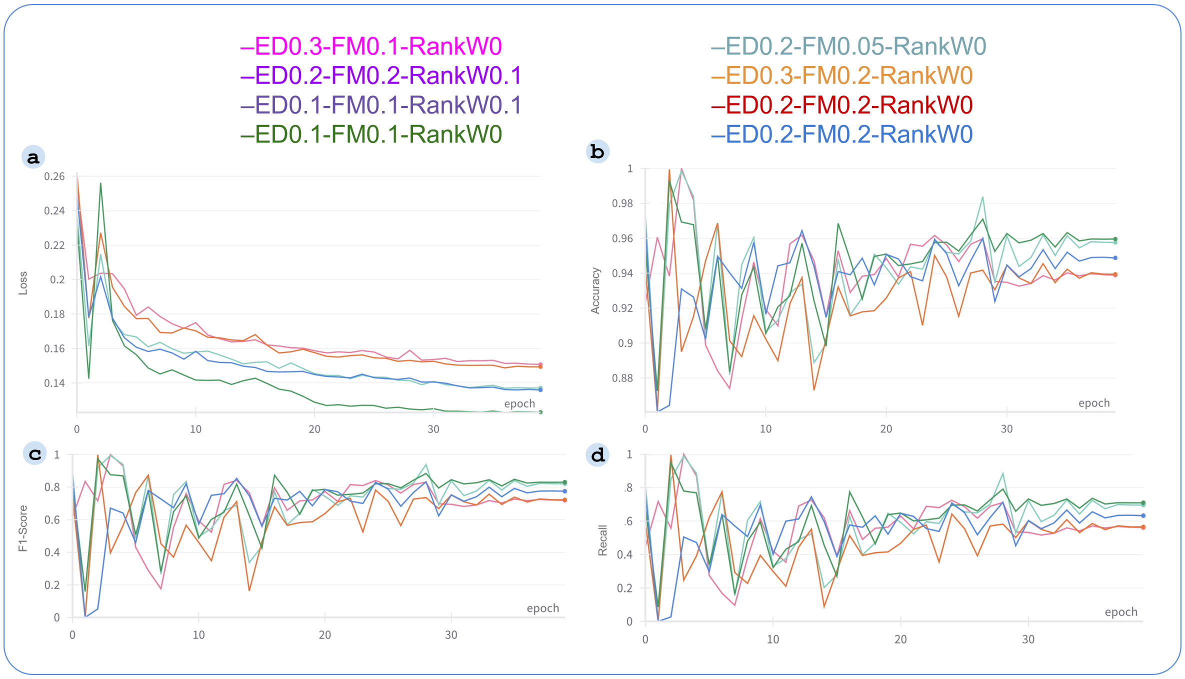

Supplementary Figure 4: Performance of `SIMBA` during supervised pretraining on simulated data. (a) Convergence of training loss over epochs. (b-d) Performance on the validation set for predicting the presence of metabolite cross-feeding presence over epochs, showing (b) accuracy, (c) F1-score, and (d) recall.

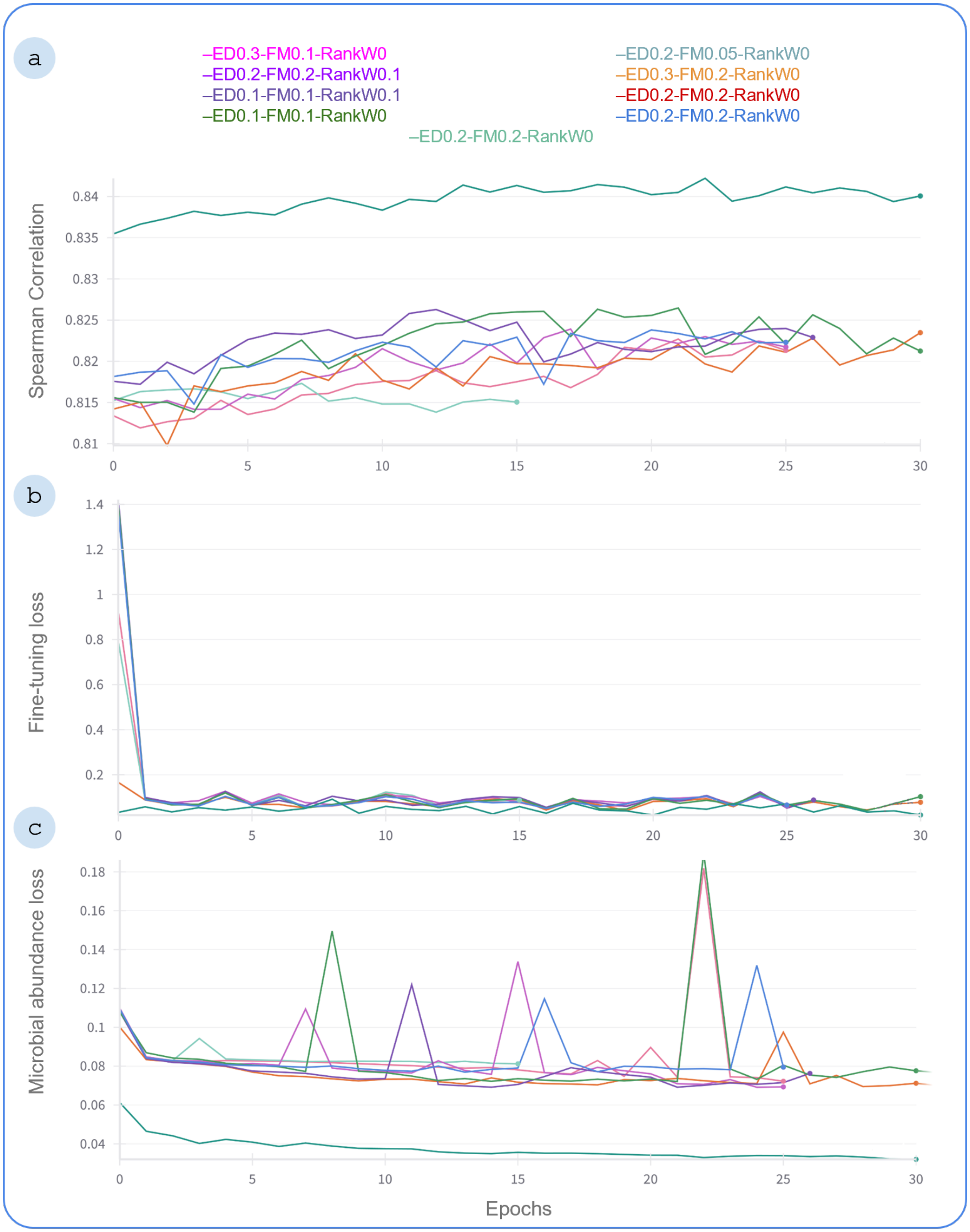

Supplementary Figure 5: Performance of SIMBA during fine-tuning on experimental data. (a) Fine-tuning validation performance of the model variants for microbial abundance prediction. Spearman correlation trajectories on the experimental validation set are shown over the course of fine-tuning epochs. (b) Convergence of fine-tuning loss over epochs. (c) Convergence of microbial abundance loss during fine-tuning.

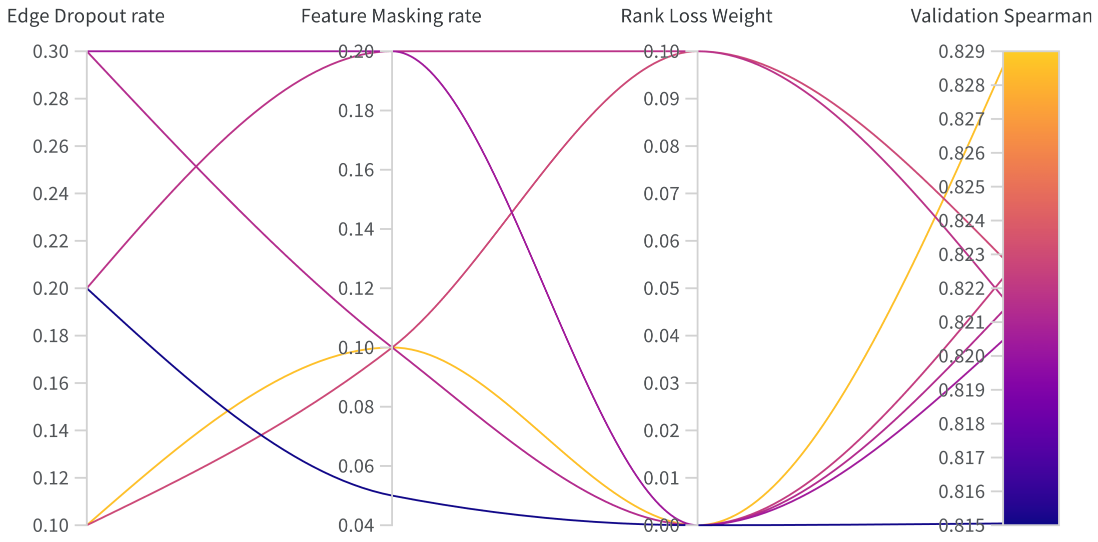

Supplementary Figure 6: Relationship between key hyperparameters and the achieved Spearman scores for microbial abundance on the validation set during Bayesian hyperparameter optimization.

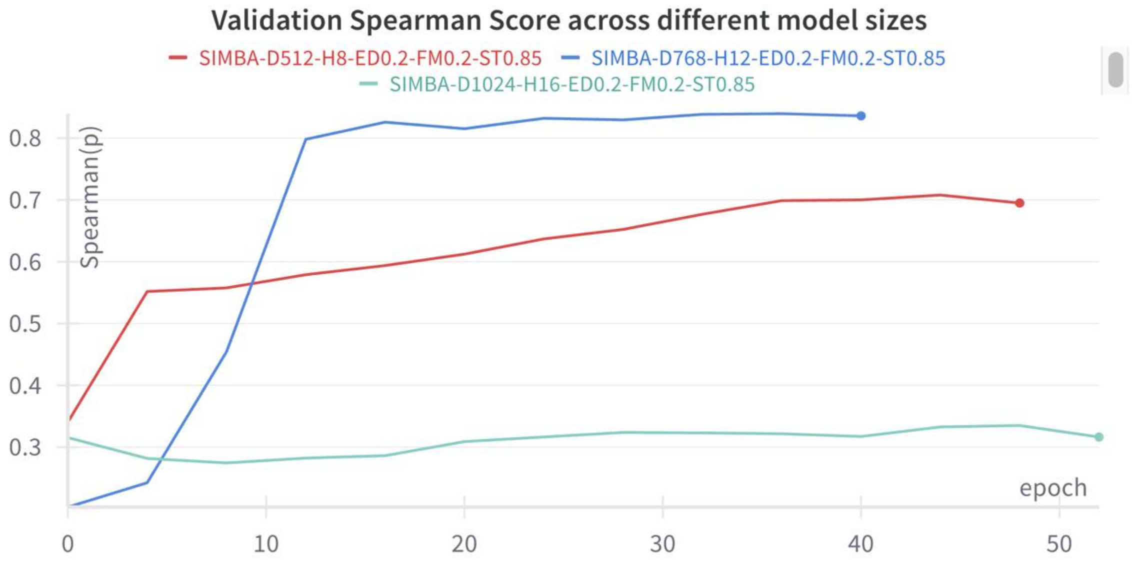

Supplementary Figure 7: Changes of Spearman scores for models with different hidden dimensions and attention heads.

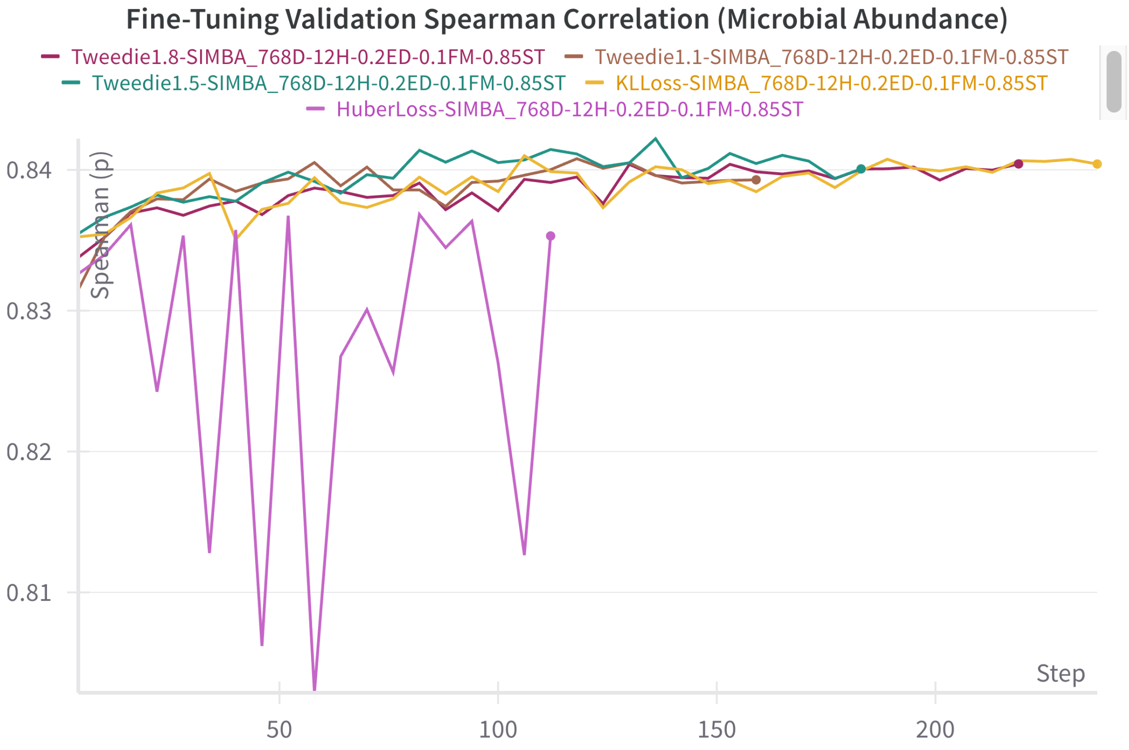

Supplementary Figure 8: Comparison study of different loss functions used for the prediction of microbial abundances based on Spearman correlation
